# Salient syllabi: Examining design characteristics of science online courses in higher education

**DOI:** 10.1371/journal.pone.0276839

**Published:** 2022-11-03

**Authors:** Christian Fischer, Peter McPartlan, Gabe Avakian Orona, Renzhe Yu, Di Xu, Mark Warschauer

**Affiliations:** 1 Hector Research Institute of Education Sciences and Psychology, University of Tübingen, Tübingen, Germany; 2 School of Education, University of California, Irvine, California, United States of America; 3 Teachers College, Columbia University, New York, New York, United States of America; Central China Normal University, CHINA

## Abstract

The importance of online learning in higher education settings is growing, not only in wake of the Covid-19 pandemic. Therefore, metrics to evaluate and increase the quality of online instruction are crucial for improving student learning. Whereas instructional quality is traditionally evaluated with course observations or student evaluations, course syllabi offer a novel approach to predict course quality even prior to the first day of classes. This study develops an online course design characteristics rubric for science course syllabi. Utilizing content analysis, inductive coding, and deductive coding, we established four broad high-quality course design categories: course organization, course objectives and alignment, interpersonal interactions, and technology. Additionally, this study exploratively applied the rubric on 11 online course syllabi (N = 635 students) and found that these design categories explained variation in student performance.

## Introduction

The importance of online coursework in higher education reached new heights due to the Coronavirus disease 2019 (Covid-19) induced shift to emergency remote education. However, even before the Covid-19 pandemic, many students enrolled in online courses. For instance, about 34% of all undergraduate students (about 5.7 million students) enrolled in at least one online course [1]. Although studies reported that online learning in higher education can have positive effects similar to face-to-face coursework [2–4], more recent studies underscored that the effectiveness of online education is a field full of heterogeneous effects with ample research studies indicating that students may struggle in online learning environments [5–9]. This may lead to lower course persistence rates in online settings, compared to their face-to-face counterparts [9, 10]. Especially, students with weaker self-regulation are challenged in online learning environments [11–14].

To unpack potential areas for improvement of online learning, recent studies examined the structure and design of online courses to identify quality indicators of online course experiences [10, 15]. Much work that examines the effectiveness of college courses relies on course observations or student evaluations [15–18]. However, course observations and student evaluations have several limitations. For instance, student course evaluations tend to disfavor female instructors [19]. In-depth course observations may provide rich information regarding course design features but require intensive time commitment from observers making data collections at scale difficult. Course syllabi may address these scalability problems as they represent a more cost-effective, less biased, and less invasive approach to examine course quality. Thus, this study utilizes course syllabi as its primary data source and adapts existing classification rubric primarily used in observational studies for course syllabi.

This study has implications for multiple stakeholders. From a theoretical perspective, it represents the first step in identifying links between syllabus-derived course design features and student performance. From a practitioner perspective, identifications of important course design features may guide instructional practice. From an administrative perspective, this study responds to the need for colleges to benchmark their online course quality as departments may want to continue including online course offering in their teaching portfolios after the Covid-19 pandemic. Inferences may support the development of cost-effective (and feasible) ways to assess many online courses and to identify courses that are not fully leveraging the affordances of online learning in a timely manner, for instance, as part of a course-level early warning system

### Online courses in college environments

The role of online education in the higher education landscape is ever increasing in the decades to come. With the shift to emergency distance learning due to the Covid-19 pandemic forcing many departments to move to fully online education, departments may choose to incorporate some courses in the regular teaching portfolio after the pandemic. Although online courses should certainly not be viewed as a one-size-fits-all solution to the future of learning, they provide some affordances that could potentially benefit learners. For example, online courses present opportunities for differentiated instruction and the ability to go at one’s own pace [20, 21]. Also, online courses allow universities greater capacity to accommodate non-traditional students who may need additional preparatory course work or flexibility of an asynchronous schedule to balance academic coursework with work and family responsibilities [22–24]. Furthermore, online education may be more cost-effective not only to universities but to students as increased access to learning opportunities may accelerate their time-to-degree [25].

Potential adversary characteristics include a reduction of social presence through fewer face-to-face interactions, which can reduce students’ motivation to carry out their study plans and the greater need for self-regulation abilities [26, 27]. Although, self-regulation is important for learning in any context, it is especially important in online learning as learner-centered approaches require greater self-directed learning skills to succeed [11–13, 28]. This has implications for both educational quality and equity. For example, institutions that enroll many students with insufficient self-regulation abilities would face greater challenges in achieving learning outcomes in online coursework. Indeed, existing studies indicate that this heterogeneity has largely been approached by investigating the context in which online courses are taught. Online courses composed of students from traditionally disadvantaged backgrounds are more likely to see worse achievement outcomes online compared to face-to-face courses [5, 29–31]. Because of this, institution type may also play a role, with negative effects coming from community college populations [8, 32], which tend enroll more students from disadvantaged backgrounds. However, less research focused on how online courses are taught and their subsequent impact of specific course elements on student success [15]. Thus, this study explores course design features that are common in online education to examine the potential quality of online course offerings.

### Design characteristics of online courses

Among studies that have investigated the quality of online courses, a handful of consistent themes emerge. Jaggars and Xu provided a comprehensive framework for understanding indicators of quality online course delivery [15]. Their review spanned research from practitioner-oriented literature, surveys of student and instructor opinion regarding elements of high-quality online courses, experimental studies manipulating specific design features, and rubrics of quality assessment developed by educational associations. The resultant framework suggested four main areas of quality, including course organization and presentation, learning objectives and alignment assessment, interpersonal interactions, and technology. This study builds on this framework but adapts it for course syllabi as its primary data source.

#### Course organization

A popular metric of online course quality, course organization, involves the clarity and consistency of the course structure. Research from opinion surveys and practitioner literature emphasize the importance of the course’s presentation to course quality, finding associations with students’ appreciation of the instructor [33–35]. Students consider “ease of use” and course organization an important criteria for determining course quality [36]. Course organization can be evident in course outlines, hyperlink structures, instructions for assignments, and grading policies [37, 38]. Creating a navigable infrastructure (e.g., organization of weekly assignments, instructions for getting started) supports student autonomy within the course, which is essential for asynchronous learning [39, 40]. Supporting students’ autonomy not only applies to their ability to find resources online, but also their ability to self-regulate their learning appropriately based on the organization of course expectations. Course organization may improve students’ experiences when it clearly conveys assignment due dates, preparation time, and policies towards late submissions [14, 15, 41].

#### Learning objectives and alignment

Especially in online learning environments, where students are expected to study more independently, carefully choosing and presenting a course’s learning objectives is important [34, 42]. Several quality rubrics highlight that learning objectives should be measurable and consistent [34, 43]. Accordingly, assessments should be aligned with those learning objectives, for instance, utilizing detailed assignment descriptions. Instructors are encouraged to align course elements with overall learning objectives by precisely stating learning objectives for which course activities then serve to measure, especially when it comes to exams [36, 39]. Within each course activity, instructors can support students’ achievement of each learning objective by suggesting strategies for successful study behavior [44]. This may include clear instructions on the sequence in which course elements should be started and completed to optimize understanding [36, 40] and to encourage students to self-test their knowledge and space their study time into multiple sessions throughout a week [45–47].

#### Interpersonal interactions

In online courses, interpersonal interactions stand out as for promoting cognitive engagement with the course and students’ psychological connection to the course [15]. Time and space between instructors and students inherent to online courses creates greater transactional distance, which may threaten students’ psychological connection to the course [48]. Instructors can counteract this by cultivating a stronger social presence, students’ perceptions that the instructor is a “real person.” In practice, this may be approached through an instructor’s use of humor and self-disclosure, both of which help convey the uniqueness of an instructor’s personality in the absence of non-verbal communication [49]. Similarly, communication that uses a tone that is friendly, open, and caring can help students feel a sense of identity with the course, as can preparing students for the hazards of discussing sensitive topics in the classroom [39, 50]. Instructors can even increase the effectiveness of student-peer interactions by providing guidelines for “netiquette,” or preparing students to use a “connected voice” when working with each other [39]. Furthermore, the effectiveness of interactions depends also their frequency and accessibility [51]. Students’ perceptions of instructor accessibility can be supported by quickly replying to email communications and facilitating social integration [39, 40]. Connecting with students through multiple digital channels, including social networking sites, can also increase instructor accessibility [49]. Students can also benefit if the instructor facilitates student-peer communication that has a clear purpose [52]. Similarly, students may benefit from voluntary peer communication, for instance, on course-specific discussion boards [53].

#### Technology

The approaches of how instructors utilize technology is associated with student satisfaction and performance [33, 36]. It is important to note that using technology for the sake of technology is not necessitating improvements for student learning as it may also induce extraneous cognitive loads [54]. Therefore, theoretical reviews have encouraged instructors to make the challenges of online learning explicit to students, along with information about the course and technical support [39, 55]. For instance, using technology to optimize the potential for learners’ autonomy is critical beyond just its mere presence [56]. “Linkability” to important resources outside the course website, for instance, increases instructors’ ability to moderate online courses effectively [57]. Similarly, easily accessible and downloadable technology are important quality course indicators, as well as tools that help students connect with the course material beyond simply reading text [33]. Furthermore, the effectiveness of technology should coincide with lowering barriers to participation so that students have sufficient access to required technology [34].

### Research questions

This study connects to the research base on online courses in higher education, ultimately attempting to support improvements of instructional practices in online settings. This study adapts and examines commonly used course design rubrics for course syllabi. In addition, we provide an exploratory empirical investigation on potential correlational associations of online course design features with student grades, which may inspire future research pursuits including testing causal processes and systematically analyzing larger databases of course syllabi. This study is guided by the following research questions (RQs):

RQ1: What are trends in course design characteristics across online courses?
RQ2: What are associations between online course design characteristics and student grades?

## Material and methods

### Study setting

This project is situated at a large public research university in California. This institution enrolls a more diverse student body compared to peer institutions in the United States holding federal designation as both an Asian American and Native American Pacific Islander-Serving Institution and a Hispanic-Serving Institution.

Data for this study was provided from multiple units on campus including Admissions, the Office of Institutional Research, and the Registrar’s Office. Course syllabi were collected through targeted emails to science course instructors (i.e., Biology, Chemistry, Physics) who taught at least one four-unit online lecture course for undergraduates between 2014 and 2017.

Instructors were asked to share their course syllabi or information about their course in the learning management system, which we treated as de facto syllabi. Notably, there were no departmental or university standards for the content of syllabi, which were solely designed by the instructors of each course. If an instructor taught the same online course multiple times, we only requested the syllabus for the latest course iteration. In total, 47 courses fit our inclusion criteria with a total of 18 unique course-instructor pairings. We collected course syllabi from 11 course-instructor pairings (61.1% response rate). That said, this institution’s overall proportion of online science courses is relatively small (3.3% online courses in 2014–2017). Notably, this study takes part before the shift to emergency distance education, in which the university did not centrally organize online courses offerings. Instead, instructors could often choose whether to offer their courses in online or face-to-face modality.

These course syllabi represent four biological science and four physics courses. None of the eligible chemistry instructors provided us with course syllabi. Topics of the biological sciences courses included cell biology and genetics (biological sciences course 1); ecosystems and evolutionary processes (biological sciences course 2); proteins, properties and pathways (biological sciences course 3); and molecular, cellular, and other structural features of the human body (biological sciences course 4). Topics of the physics courses included basic concepts of force, motion, and vectors (physics course 1); oscillations, waves, fluids, and optics (physics course 2); fundamental physics and mathematical principles to understand science fiction from scientific facts and discoveries (physics course 3); and the history and methodology to study solar systems (physics course 4).

Notably, the small course-level sample size did not impede our ability to develop the course design rubric for college science courses as occurrence of additional categories and items within categories was saturated even with a subset of these syllabi. A subsequent quantitative analysis applying the coding rubric on these 11 courses (which enrolled a total of 635 students) allows for some initial explorations on potential correlations with syllabi-derived course design characteristics. However, the limited statistical power for subsequent multi-level quantitative analysis (i.e., nesting students within courses) requires caution when interpreting associations of course design characteristics with student grades.

### Development of coding rubric

Our analysis modeled the steps of content analysis in identifying sources of data, developing categories and codes, and clustering codes in themes [58]. Content analysis considers which data are to be analyzed, how they are defined, relevant population(s), context, boundaries, and intended inferences [58, 59]. The coding rubric was developed through a six-stage process:

The *first stage* consisted of an extensive review of the online teaching and learning literature. This included an extensive search for studies investigating course design features and components. Key search words included online course design features, online course components, syllabi, coding frameworks, and online teaching and learning. Articles that exhibited topics concerning online course features, observation protocols, and coding schemes were selected for detailed review. This review was used to identity important online course design features.

In the *second stage*, we classified online course design characteristics derived from the literature search into general course design categories and subcategories in a series of meetings with the research team. For instance, categories such as interpersonal communication and peer-to-peer interaction emerged from the deliberation of the online course design literature and were vetted against seminal works in the field.

The *third stage* utilized four existing course syllabi to verify the completeness and accuracy of the coding rubric and added, removed, merged, and split course design categories and subcategories as appropriate.

The *fourth stage* consisted of the item writing process. More specifically, once the category headers for various course design features were identified, we began to develop items under each domain.

In the *fifth stage* we randomly selected two syllabi to undergo review. Four researchers on the team examined the same two syllabi to determine if items needed to be refined. Interrater reliability prior to refinement of the coding schema was at κ = 0.547, indicating moderate agreement, using the Kappa coefficient derived from Davies and Fleiss’ recommendation for a fully-crossed design with three or more coders [60, 61].

In the *sixth stage* we further refined items that exhibited low reliability to finalize the coding rubric. This led to an improved interrater reliability of κ = 0.678 representing substantial agreement [61]. Afterwards, all course syllabi were split among the four researchers who developed the coding rubric, and subsequently coded according to revised coding rubric.

### Measures

#### Qualitative syllabi measures

The coding rubric includes a total of 23 items; however, only 22 were deemed relevant for science syllabi (one item pertained to the discussion of sensitive social issues, which we found to be largely absent in the examined science courses). All items are nested within the four larger course design categories, namely (a) technology, (b) course organization, (c) learning objectives and alignment, and (d) interpersonal interactions. Notably, all individual items were coded as either categorical or dichotomous variables. None of the items are reverse-coded. Table 1 describes the full coding rubric used in this study.

**Table 1:** Coding rubric.

**Technology**	
*T1—Does the instructor provide additional support in response to challenges and affordances of online learning*?	
3	Highlights characteristics of online courses and provides suggestions for successful online course participation
2	Highlights characteristics unique to online courses but does not make recommendations on what students should do
1	No special reminder of online course characteristics (difficulties and affordances)
*T2—Does this instructor make efforts to reduce the cost of accessing course materials for students*?	
3	Instructor directly provides course materials to students for free or at lower costs
2	Instructor mentions cheaper means by which to access or purchase course materials
1	Instructor does not provide information on how to access course materials without excessive costs to students
*T3—How does the instructor enable access to technical support*?	
3	Instructor gives advice on potential technical problems such as accessing course materials (e.g., FAQ, tech support, clear steps for resolution)
2	Instructor provides information about contact persons if students encounter technical problems
1	Instructor does not provide contact information or FAQs regarding technical issues
*T4—Does the course require the use of special technology/software to access course materials (e*.*g*., *specific tools*, *downloading procedures)*? *(PLEASE NOTE*: *Course website does NOT constitute a special technology/software*.*)*	
3	No special technology/software needed
2	A single special technology/software needed
1	Multiple special technologies/software needed
N/A	Information not provided in syllabus
**Course Organization**	
*O1—How are required* *online* *course components (e*.*g*. *content materials*, *assessments*, *activities) stored in the course space*?	
3	Required online course components are located within a single website/platform
1	Required online course components are located across multiple different websites/platforms
N/A	Not clear (e.g., information not clearly provided in syllabus)
*O2—How clear is the presentation of core components/requirements of the course in general*?	
3	Very clear presentation (e.g., detailed course calendar, list of grading structure, clearly organized expectations for assignments, assignments matched with resources needed to complete them)
2	Somewhat clear (e.g., information is in the syllabus but not as straightforward to get; for instance, in a long paragraph of text and/or in different locations of the syllabus)
1	Not clear (e.g., information is not clearly found in the syllabus, though it may or may not exist on an ancillary website or document)
*O3—Does the course calendar show adjustments in routine course elements (e*.*g*., *discussions*, *weekly assignments) when introducing non-routine course elements (e*.*g*., *quizzes*, *exams*, *essays*, *final projects)*?	
3	No adjustments visible from information in the syllabus
1	Adjustments visible from information in the syllabus
*O4—What kind of “redo opportunities” does the course offer*?	
3	No redo opportunities offered/mentioned
2	Redo opportunities on routine course elements (e.g., discussions, weekly assignments) OR non-routine course elements (e.g., quizzes, exams, essays, final projects)
1	Redo opportunities on routine course elements (e.g., discussions, weekly assignments) AND non-routine course elements (e.g., quizzes, exams, essays, final projects)
*O5—Does the course offer extra credit opportunities*?	
3	The syllabus does not mention extra credit opportunities
1	The course offers some extra credit opportunities to earn additional points and/or to make-up for missed points
*O6*—Does the course accept late submissions (excluding rules for special circumstances such as doctor visits or emergencies)?	
3	The course allows late submissions without penalty
2	The course allows late submissions with penalty
1	The course does not allow/mention late submissions
**Learning Objectives and Alignment**	
*L1—How does the course promote course navigation of required assignments (e*.*g*., *how to complete requirements*, *what needs to be done*, *order to complete them)*?	
3	Thorough instructions (e.g., organized by weeks or assignment types) for sequence of when and in what order course elements ought to be completed (beyond listing of due dates)
2	Some instructions for sequence of when and in which order course elements ought to be completed (beyond listing of due dates)
1	No instructions for sequence of when and in which order course elements ought to be completed (beyond listing of due dates)
*L2—How does the course provide suggestions of successful study behavior and/or study tips (in contrast to course navigation*, *study behavior describes “good to do” behavior; examples include spacing of course elements*, *reviewing study guide before attempting quiz*, *etc*.*)*?	
3	Thorough recommendations for how students might improve the efficiency of their studying (e.g., spacing, suggested study resources, peer/instructor help)
2	Some recommendations for how students might improve the efficiency of their studying
1	No recommendations for how students might improve the efficiency of their studying
*L3—Are higher level learning objectives described in the course syllabus*?	
3	Higher level learning objectives are mentioned in the course syllabus
1	Higher level learning objectives are not mentioned in the course syllabus
*L4—How much information does the instructor provide with respect to grading*?	
3	Instructor provides in-depth details on grading with detailed information on performance expectations for assignments (e.g., specific elements of an assignment)
2	Instructor provides some details on grading with information on performance expectations for assignments (e.g., some sense of rubric used for grading)
1	Instructor provides cursory details on grading (e.g., only title for assignments)
*L5—How much information does the instructor provide with respect to the alignment of course elements (assignments*, *discussions*, *etc*.*) with overall learning objectives*? *(Raters must pay attention to multiple places on the syllabus to determine alignment*.*)*	
3	Instructor provides more than one connection of course elements with course objectives
2	Instructor provides one connection of course elements with course objectives
1	Instructor does not help students understand the connection of course elements with course objectives
**Interpersonal Interactions**	
*I1 How accessible are instructors to students*?	
3	Instructor offers pre-scheduled channels for immediate, synchronous interactions (e.g., face-to-face office hours, discussion sections hosted by instructor—must be on a regular basis)
2	Instructor offers channels for students to reach out to the instructor beyond emails (e.g., monitoring discussion boards, skype meetings)
1	Instructor offers no information about channels for interactions (e.g., is only available through emails or does not mention methods of interacting)
*I2* How timely do instructors respond to students?	
3	Instructors explicitly state the timeframe in which they will respond to student email or discussion posts
1	Instructors do not explicitly state the timeframe in which they will respond to student email or discussion posts
*I3—How does the course orchestrate required student peer-to-peer interaction*?	
3	Course requires interactions with classmates, focusing on quality of content and participation
2	Course requires interactions with classmates, focusing on participation
1	Course does not require interactions with classmates
*I4—Does the course offer opportunities for non-graded*, *voluntary student peer-to-peer interaction*?	
3	Students are afforded opportunities to interact with peers on a regular basis. (e.g., virtual/non-virtual class meetings, FAQ forums, etc.)
1	Opportunities to interact with peers are unclear
*I5—How does the course provide an etiquette for interpersonal interactions*?	
3	Sets explicit guidelines on how students are expected to interact
2	Offers suggestions (which might be implicit) of how interactions may be improved. Offers few to no explicit methods of how to enact this
1	Does not include an etiquette for interpersonal interactions
*I6—Does the instructor make any mention of how online interactions work differently than face-to-face interactions*?	
3	Sets explicit guidelines on how students are expected to interact online. Offers explanation on why interacting with other students online is different than face-to-face
1	Does not contrast online interactions with face-to-face interactions
I7—*What is the social presence of the instructor*?	
3	Indicators of instructor’s unique personality are clearly evident in syllabus presentation (humor, self-disclosure, tone, graphics give you a sense of who the instructor is) in more than two occasions
2	Indicators of instructor’s personality are present but only in 1–2 occasions
1	No indicators of instructor’s unique personality are present
*I8—Does the instructor prepare students for potentially sensitive topics/material/discourses (e*.*g*., *race*, *gender*, *politics) presented in the course (if applicable)*?*	
3	Explicitly prepares students for potentially sensitive course topics (e.g., specifies exactly which course topics will be sensitive as a forewarning to students)
2	Inexplicitly prepares students for potentially sensitive course topics (e.g., vaguely suggests some course material may be sensitive)
1	Does not specifically prepare students for potentially sensitive course topics or information not provided in syllabus

Note.

* Please note that this item is less relevant for syllabi of science courses.

The descriptive quantitative analysis used sum scores that add each item rating within each course design category. Then, these sum scores were scaled to a maximum of 100 for each category to allow for cross-category comparisons.

#### Institutional data

This study used student-level institutional variables (Table 2). Variables included a continuous variable representing students’ course grades (A+, A, A-: 4.0, 4.0, 3,7; B+, B, B-: 3.3, 3.0, 2.7; C+, C, C-: 2.3, 2.0, 1.7; D+, D, D-:1.3, 1.0, 0.7; F: 0.0). Please note that these grades represent students’ final course grades, which are often a cumulation of different assignments (e.g., quizzes, exams, homework) across the course. While most courses included information on the grading policies on their course syllabi, the specific grading policies often vary across courses and instructors as university courses do not usually employ standardized assessments to measure student performance. Additional continuous variables included SAT/ACT mathematics score, which was z-score transformed, and the years of enrollment in college. Categorical student-level variables included gender, underrepresented racial/ethnic minority status, first-generation college student status, low-income status, English language learner status, and whether a student is a transfer student.

**Table 2:** Descriptive information.

	N	Mean/Percentage	SD
Course grades	635	2.60	1.06
Female	634	62.15%	
Underrepresented minority	620	22.42%	
First-generation college student	604	43.05%	
Low-income student	626	34.98%	
English language learner	626	34.98%	
Transfer student	634	8.52%	
SAT/ACT mathematics score^†^	584	81.78	10.65
Years enrolled in college	635	2.07	1.24

Note.

^†^SAT/ACT scores are transformed to a 30–100 scale according to institutional guidelines.

#### Analytical methods

To answer RQ1, descriptive analyses illustrate the distributions of scores across every item and course design category. Also, the additive scores of course design categories were visualized with a dot chart and examined within and across courses. Finally, pairwise Pearson’s correlation coefficients between course design category scores were computed and discussed.

To answer RQ2, we used linear regression models with standard errors clustered at the course level to account for the nesting of students in courses [62]. Note that this analysis is meant to be exploratory to identify potentially interesting trends and not an attempt at rigorous hypothesis testing. In this exploratory analysis, student grades represent the dependent variable. Continuous z-score transformed course design category score variables represent the independent variables. Covariates include student demographics. Model 1 includes all institutional student-level covariates, whereas Model 2 include all institutional student-level covariates and course design category score independent variables. Of interest are both the additional percentage of variance explained by inclusion of all four aggregate course design characteristics and the associations of each course design category with student grades.

Notably, we examine the explained percentages of variance in student scores and the associations of the course design with student grades for both all students and students historically underserved in STEM college environments (i.e., first-generation college students, low-income students, female students, underrepresented minority students). Modeling assumptions were tested to verify the appropriateness of the models. For instance, the absence of multicollinearity was determined through the calculation of variance inflation factors. This study applied a Markov Chain Monte Carlo multiple imputation approach with 150 iterations and 200 imputations to address missing data in the covariates [63, 64].

## Results

### Online course design trends

#### Course organization

Descriptive information on each item for each course is presented in the appendix (S1 Table). Course organization items had exceptionally little variance. In all 11 courses (100%), course materials were stored across multiple websites or platforms including Canvas, Applia, Mastering Biology, and Smartwork (O1). Nine of 11 syllabi (82%) did not mention the availability of redo opportunities (O4), and again 9 of 11 (82%) did not mention the availability of extra credit opportunities (O5). Eight of 11 syllabi (73%) presented required course assignments in a way considered “somewhat clear,” indicating that although some combination of assignment descriptions, grade breakdowns, and calendars was present in many syllabi, a very clear presentation of assignments combining all of these was present in few syllabi (O2). Additionally, 10 of 11 syllabi (91%) did not mention adjustments of routine course elements when introducing non-routine course elements, or else did not provide sufficient information (e.g., course calendar) to portray this adjustment (O3). Ten of 11 syllabi (91%) either did not allow or mention late submissions (O6). These results suggest that online course syllabi do not vary substantially in terms of course organization. Course materials are likely distributed across multiple websites, with information about required course components presented somewhat clearly. Syllabi are unlikely to exhibit a relaxed load of smaller, routine assignments when larger assignments are due (e.g., tests, essays), and are also unlikely to mention policies regarding re-doing work, extra credit, or late submissions.

#### Learning objectives and alignment

Contrary to course organization, high levels of variability among syllabi emerged when looking at the presence of learning objectives and their alignment with required course elements (S1 Table). Whereas most syllabi gave instructions for a recommend sequence of assignments, several syllabi did provide guidance beyond listing all due dates (L1). Whereas over half of the syllabi provided thorough recommendations for how students might improve their study behavior, for instance, offering study resources or ways to decide if more preparation is needed before an exam, over a third did not offer such guidance (L2). Just over half of the syllabi mentioned higher level learning objectives, whereas the other half did not (L3). Over a third of the syllabi offered in-depth details on the overall grading and rubrics for specific assignments, whereas about half provided no more information than assignments titles and due dates (L4). Finally, roughly half of the syllabi explicitly connected course requirements with course objectives whereas the other half did not (L5). In sum, instructors present very different amounts of explicit information regarding the learning objectives of their course and their expectations for assignment completion, with some thoroughly detailing their expectations for grading and rationales for required assignments and others simply not mentioning what the goals of the course are or what to expect from the assignments therein.

#### Interpersonal interactions

Across these online course syllabi, there appeared to be consistency in some forms of interpersonal interaction, but much more heterogeneity in others (S1 Table). For example, almost all syllabi (82%) mentioned the availability of regularly scheduled interactions with the instructor such as face-to-face office hours or online office hours (I1). Similarly, almost all syllabi (91%) specified that students would have regular opportunities to voluntarily interact with peers through discussion forums or class meetings (I4). Conversely, very few syllabi (27%) explicitly stated the timeframe in which students could expect the instructor to respond to student emails or discussion posts (I2). Analysis of all 11 syllabi revealed that the possibility of preparing students for sensitive topics was “not applicable,” suggesting the topics of the course did not require discussion of sensitive topics (I8).

Other interpersonal interactions items differed across courses. Just over a half of the syllabi (55%) did not require interacting with classmates through discussion posts or groupwork (I3). Of the five that required peer interactions, four emphasized that grading would be based on quality of content and participation, rather than on participation alone. Just under half of the syllabi (45%) did not include any notes about etiquette for interpersonal interactions (I5). Of the six courses including etiquette notes, often in a section titled “netiquette,” two syllabi offered specific examples of how to interact (or not interact) with classmates. Similarly, just over half the syllabi (55%) did not explicitly mention that students should be aware of how online interactions can work differently from face-to-face interactions, whereas the remaining syllabi did (I6). Finally, instructors evoked very different levels of social presence through their syllabi (I7). Just over half (55%) gave at least some indication of the instructor’s unique personality or tone through deliberately placed punctuation (e.g., exclamation points) or text emphasis tools (e.g., bolding, capital letters, underlining). Of those six syllabi, four included only up to two examples of this, but two syllabi exuded the instructor’s tone and personality through several examples throughout the syllabi. Overall, syllabi consistently provide opportunities for interacting with the teacher and classmates throughout the term, but less consistently provide guidelines for how to interact.

#### Technology

Finally, high levels of variation appeared regarding the technological affordances in the course syllabi (S1 Table). Almost all syllabi (91%) highlighted the unique challenges and affordances of online courses in some way (T1). Seven of those 10 syllabi provided suggestions about how to specifically change behavior in online courses to increase success (e.g., the advantages of altering communication when conducted online), whereas three made only mentioned that students should prepare for difference in the online environment compared to typical face-to-face experiences. Just over half (55%) of syllabi pointed out cheaper means to access or purchase course materials (T2). Just over half (55%) provided no information on contact information or FAQs regarding technical issues (T3). Of those that did, two provided basic contact information for when problems arise, and three gave specific advice on potential technical problems. Finally, syllabi were split in mentioning specialized technologies required for the course (T4). About a third (36%) required at least two additional special software or web services on top of the course website (e.g., ProctorU, ALEKS). Another third (36%) required a single additional software or web service, whereas the remaining syllabi (27%) required only the course website. Overall, these syllabi suggest that technological requirements can be very different across courses, as well as the recommendations that instructors provide for navigating, purchasing, and troubleshooting those technologies.

### Aggregate course design characteristics

**Fig 1:**
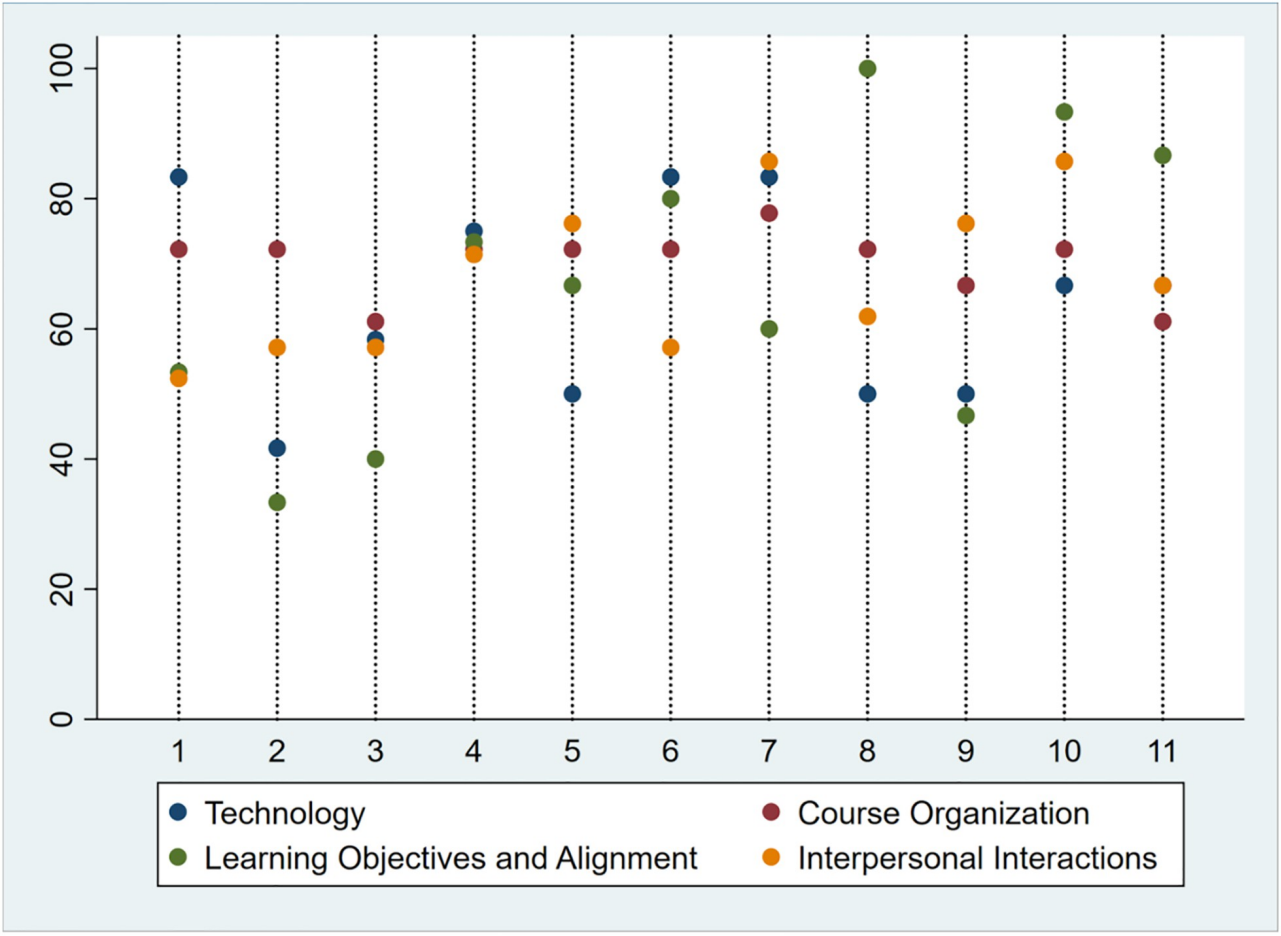
Ratings of the design characteristics by course. Please note that in a few cases, multiple categories have identical scores leading to obscured data points. In such cases, please consult the raw data in the appendix (S2 Table).

Descriptive analysis of the aggregate course design characteristics across courses indicates that highest and lowest rated design category varied across courses (Fig 1).

There seemed to be no consistent trend among design features that were higher rated in the course syllabi included in this study. For instance, whereas Learning Objectives and Alignment is the highest rated design characteristic in courses 8, 10, and 11, it represented the lowest rated design characteristic in courses 2, 3, 7, and 9. Interpersonal Interactions was the highest rated design characteristic in courses 5, 7, and 9, but the lowest rated design characteristic in courses 1, 4, and 6. Technology was the highest rated design characteristic in courses 1, 4, and 6, but the lowest rated design characteristic in courses 5, 8, and 10. Meanwhile, Course Organization was almost always between other quality categories. This large amount of course-level heterogeneity among the four quality categories is mirrored in an analysis of Pearson’s pairwise correlation coefficients (Table 3). Notably, all four categories were positively associated with each other. However, the strength of all pairwise correlations were below a moderate effect size. Notably, the correlation between Course Organization and Learning Objectives and Alignment (r = 0.131) and the correlation between Technology and Interpersonal Interactions (r = 0.039) were below recommended minimum effect sizes [65].

**Table 3:** Pairwise Pearson’s correlation of course design characteristics.

	Technology	Course organization	Learning objectives and alignment	Interpersonal interactions
Technology	1			
Course organization	0.287	1		
Learning objectives and alignment	0.228	0.131	1	
Interpersonal interactions	0.039	0.308	0.266	1

### Associations of course design characteristics with performance

Multiple linear regression models explored associations of the course design characteristics with student performance (Table 4). Subgroup analyses of students who are historically underserved in college examined potential heterogeneity in the estimates.

**Table 4:** Multiple linear regression analysis on student grades with clustered standard errors, N = 635.

	Model 1				Model 2			
	Coef	SE	t	p	Coef	SE	t	p
Intercept	2.988	0.152	19.61	<0.001	2.843	0.164	17.30	<0.001
Technology					-0.054	0.073	-0.73	0.482
Course organization					0.153	0.055	2.79	0.023
Learning objectives and alignment					-0.258	0.099	-2.60	0.030
Interpersonal interactions					0.103	0.074	1.39	0.201
Female	-0.005	0.089	-0.06	0.956	-0.034	0.064	-0.52	0.615
Underrepresented minority	-0.186	0.117	-1.59	0.149	-0.170	0.131	-1.30	0.229
First-generation college student	-0.183	0.114	-1.61	0.146	-0.149	0.096	-1.56	0.159
Low-income student	0.088	0.063	1.39	0.205	0.081	0.055	1.48	0.181
English language learner	-0.122	0.094	-1.30	0.229	-0.089	0.092	-0.96	0.364
SAT/ACT mathematics score	0.172	0.047	3.66	0.007	0.189	0.037	5.15	0.002
Transfer student	-0.239	0.217	-1.10	0.302	-0.154	0.222	-0.69	0.507
Years enrolled in college	-0.109	0.054	-2.02	0.077	-0.084	0.049	-1.73	0.120
R^2^	0.086				0.148			

The inclusion of the four course design characteristics variables in the full sample regression models led to a 6.2% increase in the explained percentage of variance of student grades (Model 1: R^2^ = 0.086, Model 2: R^2^ = 0.148). This increase in the explained percentage of variance in student grades was even greater for student groups who are historically underserved in college, with a 14.7% increase for first-generation college students (Model 1: R^2^ = 0.106, Model 2: R^2^ = 0.253), an 11.3% increase for low-income students (Model 1: R^2^ = 0.167, Model 2: R^2^ = 0.280), a 9.8% increase for female students (Model 1: R^2^ = 0.089, Model 2: R^2^ = 0.187), and a 15.2% increase for underrepresented minority students (Model 1: R^2^ = 0.094, Model 2: R^2^ = 0.246).

The regression models provided some indication of design characteristics that may be associated with student grades. In the full sample, each standard deviation increase in the Course Organization rating was associated with a 0.15 letter grade increase, *b* = 0.153, *t* = 2.79, *p* < 0.05. In contrast, each standard deviation increase in the Learning Objectives and Alignment rating was associated with a 0.26 letter grade decrease, *b* = -0.258, *t* = -2.60, *p* < 0.05. Notably, both Technology and Interpersonal Interactions course design ratings were not significantly associated with students’ grades.

Heterogeneity analysis on student groups who are traditionally underserved in college indicated similar trends. Both Technology and Interpersonal Interactions course design ratings were consistently not associated with students’ course grades across all models. Similarly, the Course Organization rating had a consistent significant association with student course grades. Each standard deviation increase in the Course Organization rating was associated with a 0.30 letter grade increase for first-generation college students, *b* = 0.298, *t* = 3.18, *p* < 0.05; a 0.24 letter grade increase for low-income students, *b* = 0.243, *t* = 4.32, *p* < 0.01; a 0.20 letter grade increase for female students, *b* = 0.199, *t* = 2.81, *p* < 0.05; and a 0.48 letter grade increase for underrepresented minority students, *b* = 0.481, *t* = 7.28, *p* < 0.001. Similarly, the Learning Objectives and Alignment rating was negatively associated with student course grades for students who are traditionally underserved in college. Each standard deviation increase in the Learning Objectives and Alignment rating was associated with a 0.34 letter grade decrease for first-generation college students, *b* = -0.341, *t* = -2.96, *p* < 0.05; a 0.36 letter grade decrease for low-income students, *b* = -0.355, *t* = -4.72, *p* < 0.01; and a 0.30 letter grade decrease for female students, *b* = -0.298, *t* = -3.08, *p* < 0.05. The appendix includes a table with all regression coefficients (S3 Table).

## Discussion

This mixed-methods study describes the qualitative development of a rubric that identifies syllabi-derived course design characteristics of college-level science online courses. This study is positioned to contribute to the research base on online learning in higher education as it represents one of the first efforts to systematically utilize course syllabi to generate inferences on the quality of online course instruction and student learning. In contrast to more resource-intensive course observations [66, 67], course syllabi and institutional data are more readily available at colleges [68–71]. Therefore, this study can provide insights on how universities could apply syllabi-based rubrics to generate inferences for educational policies, for instance, when deciding what courses to keep in an online format after the Covid-19 pandemic [72], or in an effort to use predictive analytics to enhance learning outcomes [73–75]. Instead of traditional early warning systems that intend to identify at-risk students using institutional data during a college career and/or clickstream data during a specific course [74, 76, 77], syllabi-based early warning systems would identify courses not leveraging the affordances of online learning, and could be addressed even before students are exposed to the instructional enactments. In particular, this also contrasts research assessing teaching quality through course evaluations [78]. In consequence, the three main findings are as follows:

First, course syllabi allow for an identification of online course design characteristics. In particular, it is possible to use course syllabi to detect course design categories related to Technology, Course Organization, Learning Objectives and Alignment, and Interpersonal Interactions, which were also used in observational studies [15]. Interestingly, there were no consistent trends across all courses in this study; for instance, we did not find that certain course design categories had consistently higher ratings than other design categories. In addition, the course design characteristics were not substantially correlated with each other. This indicates that these four categories can be viewed as distinct categories, which should not be collapsed in subsequent analyses.

Second, the syllabi-derived online course design characteristics can explain some variance in student grades. Although, prior performance is a considerably better predictor of student performance–as one would expect [79, 80]–it is still promising that a comparatively low-cost effort of examining course syllabi may provide additional insights in explaining student-level learning outcomes. In comparison, classroom observations are often resource-intensive while not necessarily providing valid and reliable estimates of student learning [18, 67]. Furthermore, in contrast to classroom observations, which occur during a term, syllabi can be collected before a term begins, allowing for identification of course design weaknesses before students are exposed to the course.

Third, the explorative analysis of design characteristics and student performance provides first indications that higher ratings on the Course Organization design characteristics may to be related to greater student performance. Notably, Course Organization refers to the clarity and consistency of the navigational structure, for instance, through a clear presentation of core components and requirements of the course. This finding would be in line with prior research that suggests that students tend to struggle in online courses due to lower self-regulation skills [12–14]. In courses with more transparent course organization, instructors may be able to better support their students’ self-regulatory skills [81].

### Limitations and future work

The largest limitation of this study is the assumption that the content in syllabi directly translates to instructional practice, and ultimately student learning experiences. Validation studies are highly encouraged to confirm sufficient accuracy of course syllabi compared to the current gold standard of course observations. This is mirrored in this study’s relatively large percentage of unexplained variance in student grades. Although the inclusion of online course design characteristics improved the percentage of explained variance, many potentially important constructs that relate to student learning and performance were not captured. These may include variables on student’s motivation, beliefs, and goals, self-regulation, and study skills [45, 47, 81–84]. Similarly, many teacher and teaching characteristics influence learning including teachers’ knowledge, teaching experience, and instructional practices [80, 85–89]. However, these variables are not easily available at-scale to institutions without an additional resource-intensive data collection. The goal of the paper was to utilize data that are readily available to universities without an additional resource-intensive data collection.

The second important limitation of this study is the small sample size, and thus limited statistical power [90], of examined course syllabi. While this sample size is sufficient to develop the coding rubric, inferences from the quantitative analysis need to be interpreted with caution. Robust hypothesis testing is limited due to the small statistical power as the source of variation for related hypotheses comes from between-course differences. Although this small sample size does not allow us to employ advanced quantitative modeling to thoroughly examine the impact of course design characteristics on student performance, its descriptive and exploratory nature represents a first step in the research process providing insights to inform future research.

Another limitation of this study is related to the analyzed scope of course syllabi. On the one hand, we capture a range of different aspects of constructs related to online course design. To keep the coding scheme manageable, we measure most aspects (e.g., social presence) within a construct (e.g., interpersonal interaction) with single items. A study focusing on a particular aspect of online course design (for instance, to examine the social presence in a course [91]) would need to substantially increase the item count needed to comprehensibly map the related features (e.g., also include items related to affective responses and cohesive responses related to social presence). On the other hand, this study only examined course syllabi in undergraduate online courses in science disciplines. In particular, this study reviewed online course syllabi in only biological science and physics. While it may seem reasonable to believe that similar trends would be identified in chemistry online courses [92, 93], extensions to other STEM and non-STEM disciplines are unclear. Similarly, this study was situated at a large public research university. In order to generalize findings, replication studies in other contexts with other student demographics are encouraged.

Potential future research directions that use syllabi-derived course design characteristics may ascertain whether these design characteristics help explain differences in student learning across face-to-face and online versions of the same course. The most carefully controlled study would include only courses that had the same instructor for both modalities. However, we would have to consider selection effects and the discipline of the subject. An ideal sample would intentionally balance an equal number of courses that are better and worse than their respective face-to-face counterparts. Another direction for research relates to the rise of educational data mining and learning analytics research focused on corpora of writing [69, 94, 95]. These developments have inspired researchers to use college course syllabi as a data source to better understand teaching and learning. For instance, a current research project led by Peter Bearman at Columbia utilizes machine learning, natural language processing, and social network analysis techniques to examine text corpora of hundreds of thousands of syllabi from universities all across the country to generate multidimensional measures of “liberal artsness” of student college experiences and their relations to post-graduation outcomes [96]. Similar to Peter Bearman’s work, future research could apply deep learning and machine learning algorithms on a corpus of historic course syllabi to detect underlying online course design features that are associated with student performance. Afterwards, the classification of course syllabi could be automated for any new syllabi so that this tool could serve as an early detection system for departments and administrators to identify courses and instructors that may benefit from additional institutional support. Furthermore, future research may also compare and psychometrically validate the myriad on available online course design tools (for an overview, see [97]) to help guide higher education administration on the best instruments for their individual contexts and use cases.

## Supporting information

S1 Table Raw data of course design characteristic ratings for each item and course. (DOCX)

S2 Table Raw data of aggregate course design characteristic ratings. (DOCX)

S3 Table Multiple linear regression analysis of on student grades with clustered standard errors, subgroup analyses. (DOCX)
