# Supplementary material for: Salient syllabi: Examining design characteristics of science online courses in higher education
Source: PLoS One. 2022 Nov 3;17(11):e0276839. doi: 10.1371/journal.pone.0276839 (PMC9632807; doi:10.1371/journal.pone.0276839)
Supplement: S1 Table — (DOCX) [file pone.0276839.s001.docx]

**Table S1.** Raw data of course design characteristic ratings for each item and course.

| Technology | | | | | | | | |
| --- | --- | --- | --- | --- | --- | --- | --- | --- |
| Course id | T1 | T2 | T3 | T4 |  |  |  |  |
| 1 | 3 | 2 | 3 | 2 |  |  |  |  |
| 2 | 1 | 1 | 1 | 2 |  |  |  |  |
| 3 | 2 | 2 | 1 | 2 |  |  |  |  |
| 4 | 3 | 2 | 1 | 3 |  |  |  |  |
| 5 | 2 | 2 | 1 | 1 |  |  |  |  |
| 6 | 3 | 2 | 2 | 3 |  |  |  |  |
| 7 | 3 | 1 | 3 | 3 |  |  |  |  |
| 8 | 2 | 2 | 1 | 1 |  |  |  |  |
| 9 | 3 | 1 | 1 | 1 |  |  |  |  |
| 10 | 3 | 1 | 2 | 2 |  |  |  |  |
| 11 | 3 | 1 | 3 | 1 |  |  |  |  |
| Course Organization | | | | | | | | |
| Course id | O1 | O2 | O3 | O4 | O5 | O6 |  |  |
| 1 | 1 | 2 | 3 | 3 | 3 | 1 |  |  |
| 2 | 1 | 2 | 3 | 3 | 3 | 1 |  |  |
| 3 | 1 | 2 | 3 | 3 | 1 | 1 |  |  |
| 4 | 1 | 2 | 3 | 3 | 3 | 1 |  |  |
| 5 | 1 | 2 | 3 | 3 | 3 | 1 |  |  |
| 6 | 1 | 2 | 3 | 3 | 3 | 1 |  |  |
| 7 | 1 | 3 | 3 | 3 | 3 | 1 |  |  |
| 8 | 1 | 2 | 3 | 3 | 3 | 1 |  |  |
| 9 | 1 | 3 | 3 | 2 | 1 | 2 |  |  |
| 10 | 1 | 2 | 3 | 3 | 3 | 1 |  |  |
| 11 | 1 | 3 | 1 | 2 | 3 | 1 |  |  |
| Learning Objectives and Alignment | | | | | | | | |
| Course id | L1 | L2 | L3 | L4 | L5 |  |  |  |
| 1 | 1 | 2 | 3 | 1 | 1 |  |  |  |
| 2 | 1 | 1 | 1 | 1 | 1 |  |  |  |
| 3 | 2 | 1 | 1 | 1 | 1 |  |  |  |
| 4 | 1 | 3 | 3 | 3 | 1 |  |  |  |
| 5 | 3 | 3 | 1 | 2 | 1 |  |  |  |
| 6 | 2 | 3 | 3 | 2 | 2 |  |  |  |
| 7 | 1 | 1 | 1 | 3 | 3 |  |  |  |
| 8 | 3 | 3 | 3 | 3 | 3 |  |  |  |
| 9 | 1 | 1 | 1 | 1 | 3 |  |  |  |
| 10 | 2 | 3 | 3 | 3 | 3 |  |  |  |
| 11 | 3 | 3 | 3 | 1 | 3 |  |  |  |
| Interpersonal Interactions | | | | | | | | |
| Course id | I1 | I2 | I3 | I4 | I5 | I6 | I7 | I8 |
| 1 | 3 | 1 | 1 | 3 | 1 | 1 | 1 | na |
| 2 | 3 | 1 | 1 | 3 | 1 | 1 | 2 | na |
| 3 | 3 | 1 | 1 | 3 | 1 | 1 | 2 | na |
| 4 | 3 | 1 | 1 | 3 | 3 | 3 | 1 | na |
| 5 | 3 | 1 | 2 | 3 | 3 | 3 | 1 | na |
| 6 | 1 | 3 | 1 | 1 | 2 | 3 | 1 | na |
| 7 | 2 | 3 | 3 | 3 | 2 | 3 | 2 | na |
| 8 | 3 | 1 | 1 | 3 | 1 | 1 | 3 | na |
| 9 | 3 | 1 | 3 | 3 | 2 | 3 | 1 | na |
| 10 | 3 | 3 | 3 | 3 | 2 | 1 | 3 | na |
| 11 | 3 | 1 | 3 | 3 | 1 | 1 | 2 | na |
