# Supplementary material for: Salient syllabi: Examining design characteristics of science online courses in higher education
Source: PLoS One. 2022 Nov 3;17(11):e0276839. doi: 10.1371/journal.pone.0276839 (PMC9632807; doi:10.1371/journal.pone.0276839)
Supplement: S2 Table — (DOCX) [file pone.0276839.s002.docx]

**Table S2.** Raw data of aggregate course design characteristic ratings.

| Course id | Technology | Course organization | Learning objectives and alignment | Interpersonal interactions |
| --- | --- | --- | --- | --- |
| 1 | 83.33 | 72.22 | 53.33 | 52.38 |
| 2 | 41.67 | 72.22 | 33.33 | 57.14 |
| 3 | 58.33 | 51.11 | 40.00 | 57.14 |
| 4 | 75.00 | 72.22 | 73.33 | 71.43 |
| 5 | 50.00 | 72.22 | 66.67 | 76.19 |
| 6 | 83.33 | 72.22 | 80.00 | 57.14 |
| 7 | 83.33 | 77.78 | 60.00 | 85.71 |
| 8 | 50.00 | 72.22 | 100.00 | 61.90 |
| 9 | 50.00 | 66.67 | 46.67 | 76.19 |
| 10 | 66.67 | 72.22 | 93.33 | 85.71 |
| 11 | 66.67 | 61.11 | 86.67 | 66.67 |
