# Supplementary material for: Salient syllabi: Examining design characteristics of science online courses in higher education
Source: PLoS One. 2022 Nov 3;17(11):e0276839. doi: 10.1371/journal.pone.0276839 (PMC9632807; doi:10.1371/journal.pone.0276839)
Supplement: S3 Table — (DOCX) [file pone.0276839.s003.docx]

**Table S3.** Multiple linear regression analysis of on student grades with clustered standard errors, subgroup analyses.

|  | Model 1 | | | | Model 2 | | | |
| --- | --- | --- | --- | --- | --- | --- | --- | --- |
|  | Coef | SE | t | p | Coef | SE | t | p |
| First-Generation College Students (N=260) | | | | | | | | |
| Intercept | 3.008 | 0.287 | 10.47 | <0.001 | 2.672 | 0.259 | 10.31 | <0.001 |
| Technology |  |  |  |  | -0.074 | 0.112 | -0.66 | 0.526 |
| Course organization |  |  |  |  | 0.298 | 0.094 | 3.18 | 0.012 |
| Learning objectives  and alignment |  |  |  |  | -0.341 | 0.115 | -2.96 | 0.017 |
| Interpersonal  interactions |  |  |  |  | 0.120 | 0.094 | 1.28 | 0.235 |
| Female | -0.104 | 0.178 | -0.58 | 0.575 | -0.003 | 0.143 | -0.02 | 0.984 |
| Underrepresented  minority | -0.309 | 0.174 | -1.78 | 0.112 | -0.237 | 0.171 | -1.39 | 0.201 |
| First-generation  college student | --- | --- | --- | --- | --- | --- | --- | --- |
| Low-income student | 0.138 | 0.113 | 1.22 | 0.256 | 0.094 | 0.107 | 0.88 | 0.405 |
| English language  learner | -0.058 | 0.131 | -0.44 | 0.669 | 0.024 | 0.139 | 0.17 | 0.868 |
| SAT/ACT  mathematics score | 0.207 | 0.087 | 2.36 | 0.046 | 0.248 | 0.063 | 3.92 | 0.005 |
| Transfer student | -0.159 | 0.212 | -0.75 | 0.477 | 0.028 | 0.237 | 0.12 | 0.909 |
| Years enrolled in  college | -0.185 | 0.152 | -1.21 | 0.259 | -0.108 | 0.083 | -1.31 | 0.226 |
| R^2^ | 0.106 |  |  |  | 0.253 |  |  |  |
| Low-Income Students (N=219) | | | | | | | | |
| Intercept | 3.223 | 0.276 | 11.68 | <0.001 | 2.909 | 0.274 | 10.62 | <0.001 |
| Technology |  |  |  |  | 0.009 | 0.070 | 0.13 | 0.902 |
| Course organization |  |  |  |  | 0.243 | 0.056 | 4.32 | 0.002 |
| Learning objectives  and alignment |  |  |  |  | -0.355 | 0.075 | -4.72 | 0.001 |
| Interpersonal  interactions |  |  |  |  | 0.101 | 0.064 | 1.58 | 0.151 |
| Female | 0.038 | 0.136 | 0.28 | 0.786 | 0.131 | 0.158 | 0.83 | 0.429 |
| Underrepresented  minority | -0.243 | 0.186 | -1.31 | 0.226 | -0.235 | 0.197 | -1.20 | 0.265 |
| First-generation  college student | -0.140 | 0.137 | -1.02 | 0.338 | -0.117 | 0.109 | -1.08 | 0.313 |
| Low-income student | --- | --- | --- | --- | --- | --- | --- | --- |
| English language  learner | 0.028 | 0.134 | 0.21 | 0.839 | 0.026 | 0.131 | 0.20 | 0.847 |
| SAT/ACT  mathematics score | 0.257 | 0.075 | 3.43 | 0.009 | 0.258 | 0.053 | 4.87 | 0.001 |
| Transfer student | -0.352 | 0.307 | -1.15 | 0.285 | -0.145 | 0.287 | -0.50 | 0.628 |
| Years enrolled in  college | -0.220 | 0.130 | -1.70 | 0.125 | -0.161 | 0.087 | -1.84 | 0.101 |
| R^2^ | 0.167 |  |  |  | 0.280 |  |  |  |
| Female Students (N=394) | | | | | | | | |
| Intercept | 2.990 | 0.146 | 20.47 | <0.001 | 2.751 | 0.164 | 16.81 | <0.001 |
| Technology |  |  |  |  | -0.065 | 0.086 | -0.76 | 0.470 |
| Course organization |  |  |  |  | 0.199 | 0.071 | 2.81 | 0.022 |
| Learning objectives  and alignment |  |  |  |  | -0.298 | 0.097 | -3.08 | 0.014 |
| Interpersonal  interactions |  |  |  |  | 0.071 | 0.081 | 0.88 | 0.404 |
| Female | --- | --- | --- | --- | --- | --- | --- | --- |
| Underrepresented  minority | -0.043 | 0.135 | -0.32 | 0.758 | -0.025 | 0.159 | -0.16 | 0.879 |
| First-generation  college student | -0.313 | 0.196 | -1.60 | 0.148 | -0.229 | 0.152 | -1.51 | 0.171 |
| Low-income student | 0.067 | 0.087 | 0.77 | 0.465 | 0.104 | 0.083 | 1.25 | 0.251 |
| English language  learner | -0.011 | 0.160 | -0.07 | 0.949 | 0.007 | 0.153 | 0.04 | 0.966 |
| SAT/ACT  mathematics score | 0.148 | 0.085 | 1.74 | 0.121 | 0.161 | 0.065 | 2.49 | 0.040 |
| Transfer student | -0.322 | 0.243 | -1.33 | 0.220 | -0.249 | 0.241 | -1.03 | 0.332 |
| Years enrolled in  college | -0.115 | 0.066 | -1.74 | 0.119 | -0.089 | 0.058 | -1.55 | 0.158 |
| R^2^ | 0.089 |  |  |  | 0.187 |  |  |  |
| Underrepresented Minority Students (N=139) | | | | | | | | |
| Intercept | 2.863 | 0.443 | 6.47 | <0.001 | 2.704 | 0.478 | 5.66 | <0.001 |
| Technology |  |  |  |  | -0.066 | 0.121 | -0.54 | 0.603 |
| Course organization |  |  |  |  | 0.481 | 0.066 | 7.28 | <0.001 |
| Learning objectives  and alignment |  |  |  |  | -0.180 | 0.160 | -1.12 | 0.294 |
| Interpersonal  interactions |  |  |  |  | 0.137 | 0.099 | 1.39 | 0.202 |
| Female | 0.312 | 0.209 | 1.49 | 0.173 | 0.365 | 0.227 | 1.60 | 0.146 |
| Underrepresented  minority | --- | --- | --- | --- | --- | --- | --- | --- |
| First-generation  college student | -0.457 | 0.248 | -1.84 | 0.104 | -0.381 | 0.258 | -1.48 | 0.179 |
| Low-income student | -0.026 | 0.197 | -0.13 | 0.898 | -0.088 | 0.146 | -0.60 | 0.563 |
| English language  learner | 0.065 | 0.197 | 0.33 | 0.752 | 0.172 | 0.208 | 0.83 | 0.433 |
| SAT/ACT  mathematics score | 0.122 | 0.087 | 1.41 | 0.201 | 0.202 | 0.087 | 2.34 | 0.052 |
| Transfer student | 0.025 | 0.286 | 0.09 | 0.933 | -0.020 | 0.248 | -0.08 | 0.937 |
| Years enrolled in  college | -0.178 | 0.138 | -1.29 | 0.231 | -0.116 | 0.131 | -0.89 | 0.401 |
| R^2^ | 0.094 |  |  |  | 0.246 |  |  |  |
